# Supplementary material for: EnzML: multi-label prediction of enzyme classes using InterPro signatures
Source: BMC Bioinformatics. 2012 Apr 25;13:61. doi: 10.1186/1471-2105-13-61 (PMC3483700; doi:10.1186/1471-2105-13-61)
Supplement: Addtional file 5 — The Java code to format the data files, evaluate and predict. The file enzml_java_code.tar.gz contains the Java code used to format database data to ARFF and XML formats, to execute cross and train-test (jackknife) evaluations and to record evaluation results to database. More information is included in the readme.txt file and the Javadoc files. The code can be used with a MySQL database. To use a different database software, other JDBC drivers might be required. [file 1471-2105-13-61-S5.gz › java_code/utils/doc/edu/cornell/lassp/houle/RngPack/Ranecu.html]

Ranecu


---


|  |  |  |
| --- | --- | --- |
| **Overview** \| **Package** \| **Class** \| **Use** \| **Tree** \| **Deprecated** \| **Index** \| **Help** | |  |
| **PREV CLASS**   **NEXT CLASS** | **FRAMES**    **NO FRAMES**     **All Classes** |  |
| SUMMARY: NESTED \| FIELD \| CONSTR \| METHOD | DETAIL: FIELD \| CONSTR \| METHOD |  |


---


## edu.cornell.lassp.houle.RngPack Class Ranecu

```
java.lang.Object
  cern.colt.PersistentObject
      edu.cornell.lassp.houle.RngPack.RandomElement
          edu.cornell.lassp.houle.RngPack.RandomSeedable
              edu.cornell.lassp.houle.RngPack.Ranecu
```

**All Implemented Interfaces:**: java.io.Serializable, java.lang.Cloneable

---

``` public class Ranecu extends RandomSeedable ```

Ranecu is an advanced multiplicative linear congruential random number
generator with a period of aproximately 10^18^. Ranecu is a direct
translation from Fortran of the **RANECU** subroutine published in the
paper   
F. James, Comp. Phys. Comm. **60** (1990) p 329-344   
The algorithm was originally described in   
P. L'Ecuyer, Commun. ACM. **1988** (1988) p 742   

More info .

Source code  is available.

**Version:**
:   1.0

**Author:**
:   Paul Houle  (E-mail:
    houle@msc.cornell.edu)

**See Also:**: Serialized Form

---

| **Field Summary** | |
| --- | --- |
| `static int` | `DEFSEED1`             default iseed1 = 12345 |
| `static int` | `DEFSEED2`             default iseed2 = 67890 |


**Constructor Summary**

`Ranecu()`
  
          Initialize RANECU with the default seeds from James.

`Ranecu(java.util.Date d)`

`Ranecu(int s1,
int s2)`
  
          Initialize RANECU with two specified integer seeds.

`Ranecu(long l)`


| **Method Summary** | |
| --- | --- |
| `long` | `getSeed()` |
| `double` | `raw()`             Returns a uniformly distributed pseudo-random number. |
| `void` | `raw(double[] d, int n)`             This is an inline version that returns an array of doubles for speed. |

**Methods inherited from class edu.cornell.lassp.houle.RngPack.RandomSeedable**

`ClockSeed, ClockSeed`

**Methods inherited from class edu.cornell.lassp.houle.RngPack.RandomElement**

`choose, choose, gaussian, gaussian, powlaw, raw, uniform`

**Methods inherited from class cern.colt.PersistentObject**

`clone`

**Methods inherited from class java.lang.Object**

`equals, getClass, hashCode, notify, notifyAll, toString, wait, wait, wait`

**Field Detail**

### DEFSEED1

```
public static int DEFSEED1
```

:   default iseed1 = 12345

---


### DEFSEED2

```
public static int DEFSEED2
```

:   default iseed2 = 67890


**Constructor Detail**

### Ranecu

```
public Ranecu()
```

:   Initialize RANECU with the default seeds from James.

---


### Ranecu

```
public Ranecu(java.util.Date d)
```

---


### Ranecu

```
public Ranecu(int s1,
              int s2)
```

:   Initialize RANECU with two specified integer seeds. Use this
    to introduce repeatable seeds. Equivalent to
    `Ranecu(s1*(long) Integer.MAX_VALUE)+s2)`

    **Parameters:**: `s1` - seed integer 1 (MSW): `s2` - seed integer 2 (LSW)

---


### Ranecu

```
public Ranecu(long l)
```


**Method Detail**

### getSeed

```
public long getSeed()
```

:   **Returns:**: the current generator state as a long. Can be used to restart the generator where one left off.

---


### raw

```
public final double raw()
```

:   **Description copied from class: `RandomElement`**
:   Returns a uniformly distributed pseudo-random number. The abstract method
    that must be defined to make a working RandomElement. See the class
    `RandomJava` for an example of how to do this.

    :   **Specified by:**: `raw` in class `RandomElement`
    :   **Returns:**: a uniformly distributed pseudo-random double in the range (0,1). **See Also:**: `RandomElement.raw()`

---


### raw

```
public final void raw(double[] d,
                      int n)
```

:   This is an inline version that returns an array of doubles for speed.

    :   **Overrides:**: `raw` in class `RandomElement`
    :   **Parameters:**: `d` - array to be filled with doubles: `n` - number of doubles to generate


---


|  |  |  |
| --- | --- | --- |
| **Overview** \| **Package** \| **Class** \| **Use** \| **Tree** \| **Deprecated** \| **Index** \| **Help** | |  |
| **PREV CLASS**   **NEXT CLASS** | **FRAMES**    **NO FRAMES**     **All Classes** |  |
| SUMMARY: NESTED \| FIELD \| CONSTR \| METHOD | DETAIL: FIELD \| CONSTR \| METHOD |  |


---
